# Polar Metabolite Profiles Distinguish Between Early and Severe Sub-Maintenance Nutritional States of Wild Bighorn Sheep

**DOI:** 10.3390/metabo15030154

**Published:** 2025-02-24

**Authors:** Galen O’Shea-Stone, Brian Tripet, Jennifer Thomson, Robert Garrott, Valérie Copié

**Affiliations:** 1 Department of Chemistry and Biochemistry, Montana State University, Bozeman, MT 59715, USA; 2 Department of Animal and Range Sciences, Montana State University, Bozeman, MT 59717, USA; 3 Department of Ecology, Montana State University, Bozeman, MT 59717, USA

**Keywords:** environmental metabolomics, nuclear magnetic resonance spectroscopy, multivariate statistical analysis, nutritional variance, machine learning, wildlife nutrition, conservation ecology, wild bighorn sheep

## Abstract

**Background:** Understanding the metabolic adaptations of wild bighorn sheep (*Ovis c. canadensis*) to nutritional stress is crucial for their conservation. **Methods:** This study employed ^1^H nuclear magnetic resonance (NMR) metabolomics to investigate the biochemical responses of these animals to varying sub-maintenance nutritional states. Serum samples from 388 wild bighorn sheep collected between 2014 and 2017 from December (early sub-maintenance) through March (severe sub-maintenance) across Wyoming and Montana were analyzed. Multivariate statistics and machine learning analyses were employed to identify characteristic metabolic patterns and metabolic interactions between early and severe sub-maintenance nutritional states. **Results:** Significant differences were observed in the levels of 15 of the 49 quantified metabolites, including formate, thymine, glucose, choline, and others, pointing to disruptions in one-carbon, amino acid, and central carbon metabolic pathways. These metabolites may serve as indicators of critical physiological processes such as nutritional intake, immune function, energy metabolism, and protein catabolism, which are essential for understanding how wild bighorn sheep adapt to nutritional stress. **Conclusions:** This study has generated valuable insights into molecular networks underlying the metabolic resilience of wild bighorn sheep, highlighting the potential for using specific biochemical markers to evaluate nutritional and energetic states in free-ranging ungulates. These insights may help wildlife managers and ecologists compare populations across different times in seasonal cycles, providing information to assess the adequacy of seasonal ranges and support conservation efforts. This research strengthens our understanding of metabolic adaptations to environmental stressors in wild ruminants, offering a foundation for improving management practices to maintain healthy bighorn sheep populations.

## 1. Introduction

**Figure 1:**
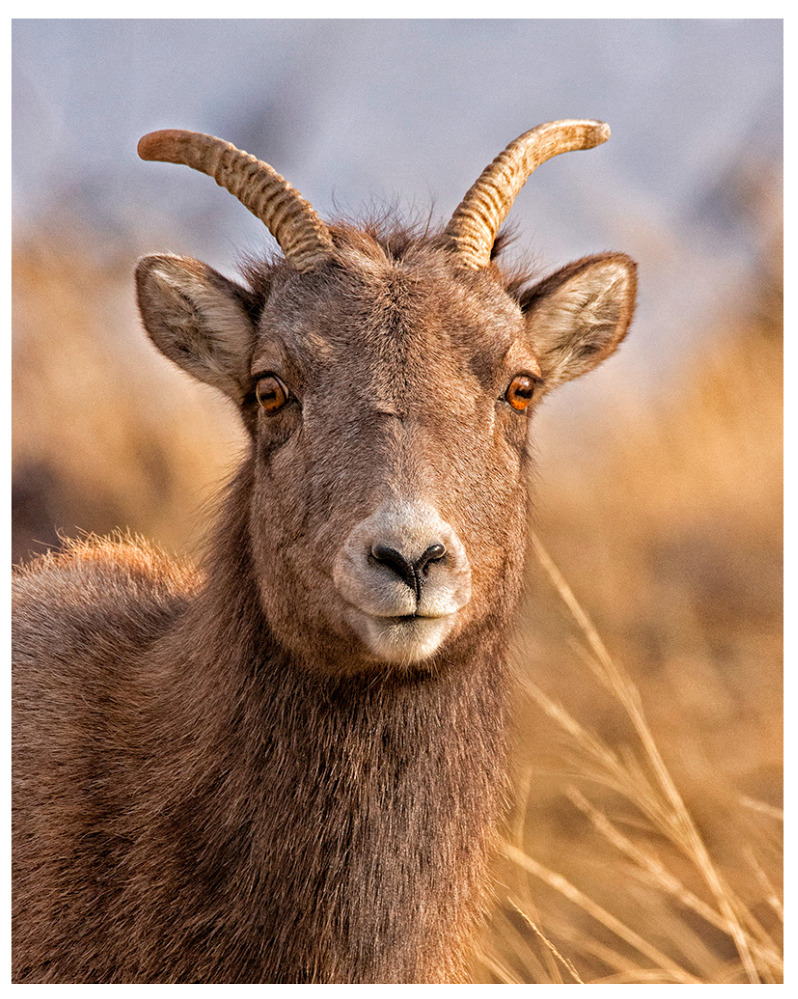
An adult female Rocky Mountain wild bighorn sheep (*Ovis canadensis canadensis*) on a Montana winter range in December, at the beginning of the winter season before the development of snowpack. The animal is likely in peak body condition with fat and lean body mass reserves accrued over the short growing season to survive the 7–8-month long season of senescent forage, cold temperatures, deep snowpack, and consequent sub-maintenance diet.

Rocky Mountain bighorn sheep (*Ovis c. canadensis*) are iconic symbols of Western North America and can survive in some of the region’s most rugged and remote landscapes (Figure 1). These wild ungulates inhabit diverse environments ranging from the arid deserts to the alpine areas of North America, where they endure substantial seasonal fluctuations in forage availability [1]. The nutritional status of these animals is a critical determinant of their survival, reproduction, and overall population dynamics. Enhancing our understanding of their metabolic responses to nutritional stress is essential [2,3]. Bighorn sheep experience pronounced metabolic and physiological changes throughout the year, driven primarily by seasonal availability, forage quality, and fundamental seasonal changes to basal metabolic rate [1,4,5]. During winter months, when forage is scarce and typically of lower nutritional value, these animals rely heavily on their body reserves, triggering a change from a maintenance dietary status to what is referred to as a sub-maintenance (SM) nutritional state [6]. These dietary deficits result in intricate metabolic adaptations that allow wild bighorn sheep to survive the extremely harsh seasonal variations associated with their habitats [7]. Fluctuations in nutrient availability have significant implications for the health and fitness of these animals throughout the year. However, little is known about the biochemical mechanisms underlying these metabolic adaptions through the seasonal changes in nutrient availability beyond increased fat and protein catabolism proportional to nutrient scarcity [8]. To our knowledge, this study is the first to characterize the nutritional adaptations of wild bighorn sheep using ^1^H nuclear magnetic resonance (NMR) metabolomics. This powerful analytical approach generates snapshots of the metabolic status of complex organisms such as wild bighorn sheep and wild ungulates [9].

Metabolomics involves systematically studying characteristic chemical fingerprints (i.e., characteristic patterns of small molecules or metabolites) engaged in specific cellular processes. Metabolomics enables a detailed identification and quantification of small molecules (typically MW < 1000 Da) present in biological samples. This approach is particularly well-suited to investigate the metabolic responses of wild animals to environmental stressors, as it allows for the detection of alterations in metabolite levels and metabolic pathway usage that may not be easily assessed quantitatively through traditional physiological or observational studies of wild animals [10]. ^1^H NMR metabolomics offers several advantages for this study, including high reproducibility, the ability to simultaneously detect a range of polar (water-soluble) metabolites, and minimal sample preparation requirements [11,12]. This study has focused on determining the polar serum metabolite profiles of wild bighorn sheep captured during the winter months (December through March) in 2014–2017 across different locations in Wyoming and Montana [10]. Through analyzing the serum metabolite profiles of these wild bighorn sheep, this study aimed to identify key metabolic pathways and metabolic interactions impacted by nutritional stress and establish specific metabolite patterns that could serve as robust indicators of the different nutritional and physiological states of these wild animals. In addition to examining the metabolic adaptations of bighorn sheep to nutritional stress, results from this study may guide wild bighorn sheep conservation strategies. Identifying metabolic indicators of the nutritional health of wild bighorn sheep could guide the development of more precise and quantitative tools to monitor and assess the nutritional status of wild bighorn sheep populations and the adequacy of seasonal ranges to support productive populations. The serum metabolomics study presented here employed multivariate statistical analyses and machine learning tools to identify and examine impacted metabolic pathways. Findings from this work have enhanced our understanding of wild bighorn sheep biology and physiological adaptations to their changing environments, including nutritional stress.

## 2. Materials and Methods

### 2.1. Animal Capture and Handling

Procedures for animal capture, handling, permit information, and sample preparation for this work have been reported in a previous publication [10].

### 2.2. Experimental Design and Study Animals

Serum samples were obtained from a total of 388 adult female wild bighorn sheep in Wyoming (7 locations) and Montana (4 locations) via helicopter netgun capture techniques, as described previously [10]. The animal captures occurred from December to March in the winters of 2014–2015, 2015–2016, and 2016–2017. Due to seasonal changes, these wild animals were sustained on senescent native forages, resulting in sub-maintenance diets from late summer/early fall until the onset of the growing season the following spring. Animals captured in December months (*n* = 170) were classified as “early sub-maintenance” (early-SM); animals captured in January (*n* = 35) were classified as “moderate sub-maintenance” (mod-SM), and animals captured from February through March (*n* = 183), which subsisted approximately 6 months on senescent forages, were classified as “severe sub-maintenance” (severe-SM).

### 2.3. Dataset Curation and Exploratory Analysis

Due to the uneven number of samples per group that led to an unbalanced dataset of metabolite concentrations between the early, moderate, and severe groups, a sensitivity analysis was conducted to evaluate the impact of the smallest sample group (mod-SM, *n* = 35) on the overall analysis. This analysis involved comparing the mean metabolite concentrations across all groups (early-SM, mod-SM, severe-SM) with the means recalculated following the exclusion of the mod-SM group (Supplementary Figure S1). This analysis determined that the inclusion or exclusion of the mod-SM group did not significantly alter the pattern of changes observed in the metabolic profiles of the early-SM and severe-SM. Furthermore, including the mod-SM group with such a small sample size can result in skewed conclusions due to insufficient data to support statistical power and increasing type II errors [13]. PCA analysis indicated that the mod-SM group did not form a distinct metabolic cluster but instead overlapped with both early-SM and severe-SM groups, suggesting that it possibly represents a transitional rather than a metabolically distinct state. Excluding the mod-SM group ensures that metabolic differences observed between the early-SM and severe-SM groups are driven by biological variation rather than sample size imbalances.

Due to these factors, the decision was made to exclude the mod-SM group for the remainder of the analysis (Figure S1). Additionally, upon initial exploratory analysis using Principal Component Analysis (PCA), a small subset of 10 serum samples that were sequentially collected were found to be lacking a substantial (>20%) number of metabolites and associated concentration values; these 10 samples were, therefore, excluded [14,15].

Most of the samples collected came from unique animal capture events. However, due to research protocols for two of the Wyoming herds, several animals (37/351) were repeatedly sampled during a given year and/or across multiple years, resulting in 115 of 338 serum samples that could not be classified as independent sampling events (Supplementary Table S1) [10]. To assess the impact of these repeated measurements on the global analysis of the entire dataset of metabolite concentrations, custom code was written in Python version 3.11.8 to perform a random selection of a unique sample from each animal to remove the inclusion of repeated measurements on the same animal in the dataset. Following the selection of a unique sample set (i.e., selection of a single sample per animal), a visual inspection of the distributions was conducted to evaluate whether the sample selections affected the outcome analysis of a randomly selected subset of 12 metabolites. This analysis involved comparing the data distributions in the original complete dataset (assembled from single and repeated animal captures) with those of the modified dataset (originating from single animal capture events), using histograms, density plots, and PCA analysis (see Supplementary Figure S2). To quantitatively assess the distribution differences in metabolite concentrations between the original and modified datasets, an exploratory PCA analysis and the Kolmogorov–Smirnov (KS) test for individual metabolites were employed [16,17]. Analyses were conducted using Python’s scipy package (version 1.41.1), with the ks_2samp function for the KS test [18]. These metrics facilitated a quantitative evaluation of how dataset modification and selection of a unique sample set for each animal influenced the distribution properties of the metabolite profiles (Figure S2). This evaluation, along with a visual inspection of the data distribution of the original and modified metabolite concentration tables, indicated that removing samples from repeated animal captures had a minimal impact on the overall results. This analysis, thus, supported the statistical benefit of preserving repeated sample collections on the same animals and using the original metabolite concentration dataset for the remainder of the analysis. Including all serum samples yielded enhanced statistical power and improved statistical model accuracy of resulting observed metabolite patterns and best utilized the valuable information obtained from this expensive, challenging, and extensive wild bighorn sheep capture study.

### 2.4. Polar Metabolite Extraction and NMR Data Acquisition

Extraction of polar metabolites and collection of 1D ^1^H NMR spectra were performed as described in [10,19].

### 2.5. Spectral Analysis and Statistical Analyses

Spectral analysis, processing, and metabolite annotations were performed using the Chenomx NMR Software (Version 8.4; Chenomx Inc., Edmonton, AB, Canada), as described in previously published studies [10,20], and resulted in the identification and quantification of 49 water-soluble metabolites. Metabolite concentrations (in μM) were exported as .csv files for further analysis.

Metabolite concentration data were preprocessed and analyzed using in-house computer scripts developed in Python. Preprocessing involved imputing missing values, which replaced one-fifth of the minimum positive value detected for each metabolite, ensuring minimal impact on the overall data distribution [21]. Following imputation, a log transformation was applied to the entire dataset to correct heteroskedasticity [22]. Following logarithmic transformation, metabolite concentrations were autoscaled, i.e., the data were mean-centered and divided by the standard deviation for each quantified metabolite [22]. Data visualization to evaluate the accuracy of the preliminary and post-processing stages of the analysis was accomplished by examining the range of metabolite concentrations in boxplots and density plots of a subset of all the metabolites identified using in-house Python script. This task was performed to ensure that the preprocessing steps, including imputation, log transformation, and autoscaling, did not introduce biases or distortions in the overall distribution of metabolite concentrations across the experimental groups. All statistical analyses were conducted using in-house computer scripts written in Python code (version 3.11.5) [23], leveraging libraries such as pandas (version 2.2.3) for data manipulation [24], numpy (1.26.4) for numerical computations [25], scikit-learn (version 1.6.1) for machine learning tasks [26], and matplotlib (version 3.9.3) for visualization [27]. The level of significance (α), the critical value used to determine the significance of model components and their ability to discriminate between groups or classes in the data for all tests, was set at α = 0.05 [28].

### 2.6. Multivariate Statistics and Machine Learning

#### 2.6.1. Principal Component Analysis

**Figure 2:**
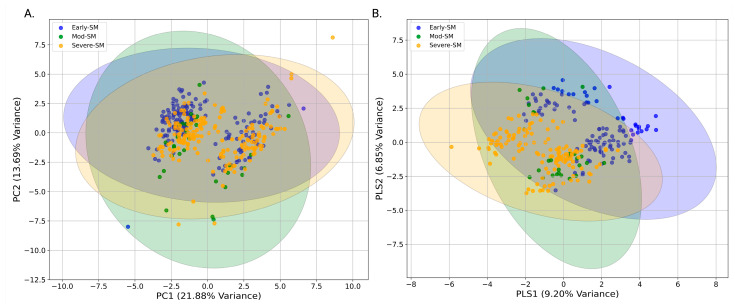
Principal Component Analysis (PCA) And Partial Least Squares Discriminant Analysis (PLSDA) of Serum Metabolic Profiles for the Three Different Sub-Maintenance Animal Groups. (**A**) The 2D-PCA scores plot represents the clustering of the different samples. Yellow dots represent serum metabolite profiles obtained from early-SM sub-maintenance animals, green from mod-SM animals, and blue from severe-SM. In this PCA analysis, PC1 and PC2 account for 21.88% and 13.48% of the variance, respectively. Shaded ellipses correspond to the 95% confidence intervals for each group. (**B**) The 2D-PLSDA score plot demonstrating the extent of the separate clustering of the early (yellow), moderate (green), and severe (blue) sub-maintenance groups based on their characteristic serum metabolite profiles, with components 1 and 2 accounting for 8.84% and 7.43% of the variance, respectively.

Principal Component Analysis (PCA) was performed as an initial exploratory data analysis (EDA) to assess the variance within the dataset and determine whether unsupervised clustering could distinguish between sub-maintenance nutritional states. The analysis was carried out using an in-house Python script with data preprocessed as described previously to normalize metabolite concentrations [24,25,26,27]. PCA was conducted, and the resulting scores plot visually represented the metabolic variation across the experimental groups (Figure 2A,B). This overlap suggested that while some metabolic differences exist between the nutritional states, these are not fully captured by unsupervised methods, prompting supervised approaches such as Partial Least Squares Discriminant Analysis (PLS-DA).

#### 2.6.2. Partial Least Squares Discriminant Analysis

Following the statistical normalization described above, the metabolite concentration data were imported into a custom-built Python-based analytical pipeline for further processing and analysis. Partial Least Square-Discriminant Analysis (PLS-DA) was employed to assess the correlation between metabolite profiles (i.e., predictors) and sample groups (i.e., responses) [28]. The optimal number of latent variables (components) needed to assess the quality of the PLS-DA model was determined by examining the cross-validated R² score that represents the proportion of variance of the sample groups inferred from the metabolite profiles [28]. PLS-DA modeling was built using the ‘PLSRegression‘ class from the scikit-learn library, configured with an optimal number of components, which was calculated to be 5 [26]. This model was split into test and training data, with the performance and significance evaluated through permutation testing and cross-validation. The predictive ability of the PLS model was assessed using 1000 random permutation steps and the resulting *p*-value. The predictive performance of this model was evaluated using confusion matrices, receiver operating characteristic (ROC) curves, R² and Q² scores on both the training and test datasets [29]. These metrics aided in quantifying the predictive relevance of this model and reflected its ability to accurately predict unseen data based on cross-validation (Supplementary Figure S2) [28,29,30].

#### 2.6.3. Univariate

The Mann–Whitney–Wilcoxon test examined metabolite levels across distinct groups within our dataset. This non-parametric test was chosen due to its effectiveness in analyzing non-normal distributions of continuous data [31]. Following the initial analysis, the Benjamini–Hochberg procedure was used to adjust for a false discovery rate (FDR) of less than 0.5, ensuring that our findings remained statistically robust amidst multiple comparisons [32,33]. Additionally, fold change (FC) analyses were conducted on the original data before column-wise normalization (i.e., log transformation and scaling), and significant features were identified as those whose fold change values exceeded (either up or down) an FC threshold set at FC = 2.0. FC was derived from the ratios of the mean metabolite of the early-SM versus severe-SM groups, i.e., (early-SM/severe-SM) ratios. Volcano plot visualization was performed using Python and combined the results from the univariate analysis (x-axis), along with fold changes (y-axis), with respective thresholds of *p* < 0.05 (FDR corrected) and FC > 2.0 [34,35].

#### 2.6.4. ANOVA (2-Way)

Two-way Analysis of Variance (ANOVA) was performed using in-house code written in Python and permitted assessing the effects of nutritional state and capture environment on metabolite concentration patterns. *p*-values were adjusted using the Benjamini–Hochberg procedure to account for a False Discovery Rate of <0.5. Significant metabolites were identified based on adjusted *p*-values, and concentration differences were visualized to assess the differential impacts of two independent variables, i.e., nutritional state and capture environment (prairie: *n* = 88, or mountain: *n* = 310), on measured metabolite concentrations [36].

## 3. Results

### 3.1. Multiclass PCA and PLS-DA Analysis of Early, Moderate, and Severe Sub-Maintenance

**Figure 3:**
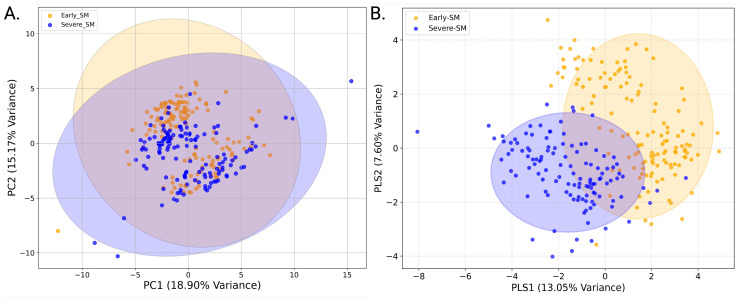
Principal Component Analysis (PCA) and Partial Least Squares Discriminant Analysis (PLS-DA) of the Serum Metabolic Profiles of Early vs. Severe Sub-maintenance Groups. (**A**) 2D-PCA scores plot showing the unsupervised clustering of each group. Orange represents the early SM group, while blue denotes severe SM. PC1 and PC2 account for 18.90% and 15.71% of the variance, respectively. Shaded ellipses correspond to the 95% confidence intervals for each group. (**B**) A 2D-PLSDA shows the distinct separation of the two groups between early(orange) and severe (blue) sub-maintenance groups. The variance explained by each component is indicated on the axes (PLS1: 13.05%, PLS2: 7.60%).

Principal Component Analysis (PCA) investigated whether measured serum metabolite levels could differentiate early sub-maintenance animals from moderate or severe sub-maintenance groups. The 2D (Figure 2A) and 3D (Figure 2B) PCA score plots indicated that this analysis did not completely distinguish the three different animal nutritional groups. Ellipses representing the 95% confidence intervals for each group indicated significant overlaps of the three groups in the 2D PCA scores plot (Figure 2A). To explore the extent of potential differences in serum metabolite levels between early, moderate, and severe sub-maintenance groups, a supervised Partial Least Squares Discriminant Analysis (PLS-DA) of resulting metabolite profiles was conducted. A 2D PLS-DA score plot (Figure 2B) indicated a slight separation between the three animal groups, with the moderate sub-maintenance(mod-SM) group cluster bridging the early-SM and severe-SM groups, as shown by the clustering of the mod-SM group in the central region of the 2D-PLS-DA score plot. It was observed that each of the sample groups formed a distinct cluster, suggesting that the PLS-DA model differentiated, to some degree, between the severity stages of nutritional sub-maintenance. However, examining the validation metrics associated with the PLS-DA model suggests that this model is overfitting and leads us to conclude that based on both PCA and PLS-DA analyses, the three different animal nutritional groups cannot be separated from each other according to their characteristic water-soluble serum metabolite profiles. (Training Q2 Score: 0.71; Training R2 Score: 0.71; Test Q2 Score: 0.56; Test R2 Score: 0.56; Permutation Test *p*-value: 5e-04). Therefore, the three-group comparison was not used for further analysis.

### 3.2. Unbalanced Sample Size Considerations

The experimental design of this study resulted in an unbalanced sample size concerning the moderate-SM group, which consisted of only 23 serum samples, a starkly lower number of samples compared to the early- and severe-sub-maintenance groups, which were comprised of 149 and 136 wild bighorn sheep serum samples, respectively. To ensure methodological rigor and statistical robustness, it was, thus, concluded that the exclusion of the moderate sub-maintenance (mod-SM) group for deeper analysis was warranted. The considerable sample size disparity between mod-SM and the other sub-maintenance nutritional groups presented a possible significant challenge, potentially introducing biases, reducing the statistical power of this study, and risking violating the assumptions underlying statistical analyses designed for balanced datasets, should the mod-SM dataset remain included. Removing this group from PCA and PLS-DA analyses mitigated the risk of diluting the significance and validity of statistical inferences drawn from analysis of the early-SM and severe-SM data analyses and ensured that resulting findings were not skewed by inconsistencies arising from unbalanced animal group sample sizes (Supplementary Figure S1).

### 3.3. PCA and PLS-DA Analysis of Early vs. Severe Sub-Maintenance

**Figure 4:**
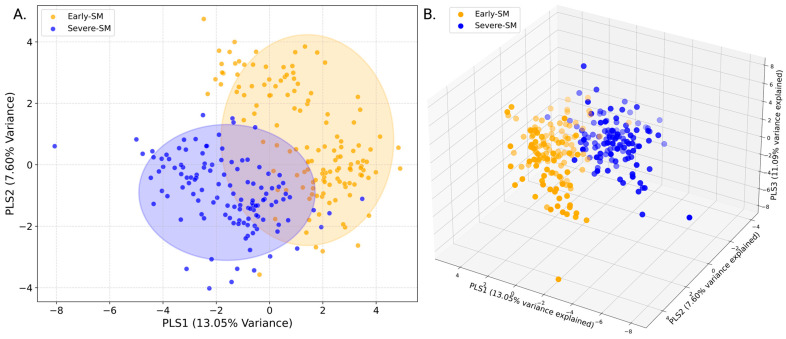
Partial Least Squares Discriminant Analysis (PLS-DA) of Early versus Severe Sub-maintenance Animal Groups. (**A**) A 2D PLS-DA score plot of serum profiles obtained from early (early-SM, yellow) and severe sub-maintenance (severe-SM, blue) animal groups. The variance accounted for by each component of the PLS-DA model is indicated on the axes (PLS1: 13.1%, PLS2: 7.6%). Blue and orange ellipses represent 95% confidence intervals. (**B**) A 3D PLS-DA score plot of early versus severe sub-maintenance groups with PLS 1–3 accounting for ~27% of the variance (PLS3: 6.1%). The positioning of the early-SM and severe-SM groups within the 3D score plot indicates the degree of metabolomic difference between the two stages of sub-maintenance. These models were cross-validated, and a permutation test was applied to ensure model robustness (see Supplementary Figure S3).

Principal component analysis was used as a first approach to assess whether early-SM and severe-SM nutritional groups could be distinguished based on their distinct polar serum metabolome profiles. The PCA analysis indicated a slight separation between the two. However, cluster overlap remained and illustrated that the two nutritional groups were not sufficiently distinct to be fully differentiated in an unsupervised PCA analysis (Figure 3A). The modest separation seen in the PCA scores plots thus motivated the application of supervised multivariate statistical analyses, including Partial Least Squares Determinant Analysis (PLS-DA). A 2D PLS-DA score plot (shown in Figure 3A,B) indicates a clear separation between early-SM (orange) and severe-SM (blue) animal groups based on their distinct and characteristic polar metabolite profiles. While a clear separation between the animal groups was observable, the PLS-DA scores plot also indicated a region of group overlap, suggesting the presence of shared metabolite patterns between early and severe sub-maintenance nutritional status. To further assess the extent of the group differences revealed in the 2D PLS-DA and to examine the sample distribution in each group cluster, a 3D PLS-DA scores plot was constructed, which included the first three principal components, highlighting a more apparent separation between the early-SM and severe-SM based on their distinct serum polar metabolomes (Figure 4B).

### 3.4. Variable Importance in Projection (VIP) Scores for Metabolites

**Figure 5:**
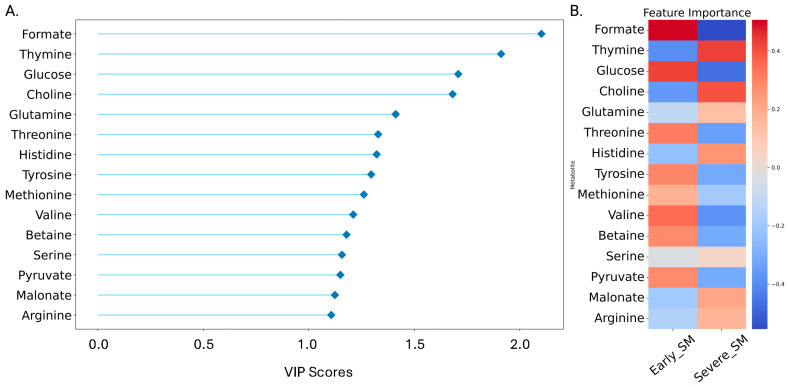
Characteristic Metabolite Level Differences between Early and Severe Sub-maintenance Groups as Revealed by PLS-DA VIP Scores Plots and Heatmap Visualization. (**A**) VIP Scores Lollipop Plot visualizes the importance of each metabolite to the PLS-DA model as determined by their VIP scores, with metabolites arranged in descending order of VIP score. Each circle (lollipop) represents a metabolite, where the line length and the circle’s position (lollipop) indicate the metabolite’s VIP score. Higher VIP scores indicate a greater contribution to this model, enabling the identification of metabolites with the most discriminative power. For this analysis, VIP scores >0.8 were considered discriminative and are included. (**B**) Heatmap visualization of relative metabolite levels for the most metabolites (as identified by VIP scores) across different groups. Each row represents a metabolite, and each column represents a group, with the color intensity indicating the metabolite’s average level within that group, ranging from red (lower levels) to dark blue (higher levels). Values in the heatmap represent each animal group’s mean metabolite concentrations.

PLS-DA results were employed to assess the discriminating power of metabolite level differences contributing to the separate clustering of the early-SM and severe-SM groups, using variable importance in projection (VIP) scores, which ranged in values from less than 0.5 to greater than 2.0 (Figure 5A). Serum metabolites with VIP scores greater than 1.0 were considered significant and included formate, thymine, glucose, choline, glutamine, threonine, and histidine (i.e., VIP scores >1.3), indicating their significant contribution to the distinction between early-SM and severe-SM nutritional states of these wild bighorn sheep. Additional serum metabolites with VIP scores greater than 1.0 included tyrosine, methionine, valine, betaine, serine, pyruvate, malonate, and arginine.

### 3.5. Heatmap of Metabolite Levels

Heatmap visualization of PLS-DA results complemented the PLS-DA VIP score analysis and reported on the relative concentrations of each metabolite in early SM versus severe SM nutritional groups (Figure 5B). This heatmap demonstrated that the formate levels are significantly higher in the early-SM group than in the severe-SM group. Additionally, concentrations of glucose, threonine, tyrosine, methionine, valine, betaine, and pyruvate were higher in the early-SM group compared to the severe-SM group, while the serum concentrations of thymine, choline, glutamine, histidine, serine, malonate, and arginine were higher in the severe-SM group compared to the early-SM group (Figure 5B). These metabolite patterns corroborated the VIP score data, supporting the findings that early and severe SM nutritional states represent significantly distinct metabolic states, which can be clearly distinguished based on distinct serum polar metabolite patterns.

### 3.6. Univariate Analysis Results

**Figure 6:**
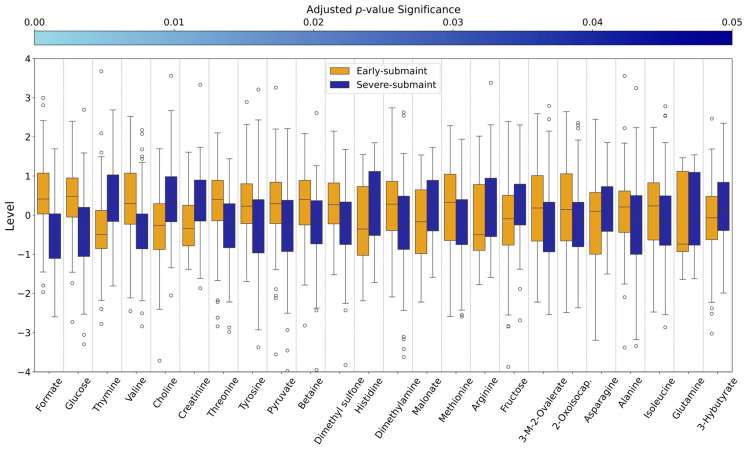
Comparative Analysis of Metabolite Levels Measured in the Early and Severe Sub-maintenance Animal Groups.

Univariate analysis employing a Mann–Whitney U test with False Discovery Rate (FDR) correction, complemented by volcano plot analysis, also identified significant differences in serum polar metabolite profiles of early-SM versus severe-SM nutritional states in wild bighorn sheep. These analyses provided additional insights into different metabolic changes in the wild bighorn sheep in response to the varying degrees of nutritional strain. Quantitative assessments of metabolite level differences between early-SM and severe-SM are reported in Supplementary Table S2 and depicted in Figure 6. Most noteworthy, formate levels were substantially altered, i.e., higher in early-SM compared to the severe-SM nutritional state, with the early-SM group having approximately 1.1 standard deviations higher concentration compared to the severe-SM (level difference of 1.06) and an extremely low adjusted *p*-value of 1.5e-18, indicating strong significance. Other metabolites with relative concentrations higher in the early-SM group included glucose, valine, threonine, and tyrosine, with positive level differences greater than 0.6 standard deviations from the mean.

Furthermore, levels of pyruvate, betaine, dimethylamine, dimethyl sulfone, 2-oxoisocaporate, 3-methyl-2-oxovalerate, malonate, methionine, isoleucine, and alanine were also elevated in the early-SM group compared to the severe-SM group, with positive differences in relative abundance ranging from 0.3 to 0.59 (Supplementary Table S2). Metabolites whose levels exhibited an opposite trend, i.e., higher in the severe-SM and lower in the early-SM group, included thymine and choline, which were among the metabolites with the most significantly lower concentrations in the early-SM compared to severe-SM groups. Thymine exhibited a concentration difference of −0.82 and an adjusted *p*-value of 3.1e-12, while the choline level difference amounted to −0.80 and an adjusted *p*-value of 2.3e-09 when examining their concentrations in early-SM versus severe-SM. Metabolites with lower serum concentrations in the early SM group also included creatinine, histidine, asparagine, malonate, fructose, arginine, 3-hydroxybutyrate, and glutamine, with level differences ranging from −0.56 to −0.24 when comparing their concentration ratios in early-SM versus the severe-SM (Supplementary Table S2).

Mann–Whitney U test results show the comparative analysis of metabolite levels between the two distinct groups, Early SM (orange/brown) and Severe SM (blue), across various metabolites, which were identified as significant through statistical testing. Metabolite levels are depicted using boxplots, with each boxplot representing the distribution of levels within each group for a specific metabolite and a light grey line between each. The y-axis labeled “Level” represents the relative metabolite abundance, i.e., how many standard deviations the log fold concentration of a metabolite is above or below the log-transformed concentration across all samples. Metabolites are ordered according to their significance levels, from the most to the least significant, as indicated by adjusted *p*-values depicted at the top of the box plots by an “adjusted *p*-value significance color scale which represents *p*-values ranging from *p* < 0.00001 (pale blue) to *p* < 0.05 (dark blue)”.

### 3.7. Volcano Plot Analysis

Table 1 summarizes identified metabolites with significant concentration changes, as assessed by volcano plot analysis. This table reports fold changes (FC), log_2_-transformed fold changes (log_2_ (FC)), adjusted *p*-values (*p*-adjusted), and negative log_10_ of the adjusted *p*-values (−log_10_ (p)) for each metabolite entry. Altogether, these parameters present a more comprehensive view of metabolite level changes observed. Formate displayed the most pronounced change with a fold change in concentration of 5.1 (i.e., significantly elevated concentration in the early-SM compared to the severe-SM group), corresponding to a log_2_ (FC) of 2.3. This log_2_ value indicates that the concentration of formate is more than 4× higher in the early-SM group, reflecting a substantial reduction in the severe-SM group. The statistical robustness of this change is underscored by an exceptionally low adjusted *p*-value of 1.3e-17. Similarly, glucose concentrations were higher in the early-SM group than the severe-SM, as evidenced by a fold change of 2.8 and a corresponding log_2_(FC), indicating a near-tripling in glucose levels in the early-SM group. The corresponding decrease in glucose levels in the severe-SM group was statistically significant, with an adjusted *p*-value of 2.3e-17. Other metabolites with noteworthy higher concentrations in the early-SM group included valine, with a log_2_ (FC) of 1.7 and a p.adj. of 2.4e-11, threonine, log_2_ (FC) of 1.3 and p.adj. of 2.6e-10, 2-oxoisocaproate, log_2_ (FC) of 1.2 and p.adj. of 2.0e-04. Betaine and dimethyl sulfone, both with a log_2_ (FC) of 1.0, with adjusted *p*-values of 2.9e-6 and 3.3e-06, respectively. Conversely, several metabolites were higher in concentration in the severe-SM group (i.e., lower concentration in the early-SM group), as indicated by negative log_2_ (FC) values. Thymine showed the largest change with a log_2_ (FC) of −1.9 and a highly significant p.adj. of 1.2e-15. Creatinine and choline were also lower in concentration in the early-SM group, with log_2_ (FC) values of −1.6 and −1.41 and adjusted *p*-values of 4.7e-11 and 1.1e-10, respectively. These log_2_-transformed fold changes provided a logarithmic scale to better evaluate exponential changes in metabolite concentrations between early- and severe- sub-maintenance nutritional status. These data further underscore the broad range of metabolite changes occurring when comparing early-SM and severe-SM nutritional groups of wild bighorn sheep as forage availability decreases over the three-month winter periods.

### 3.8. Multiple Factor Analysis: Two-Way ANOVA

**Figure 7:**
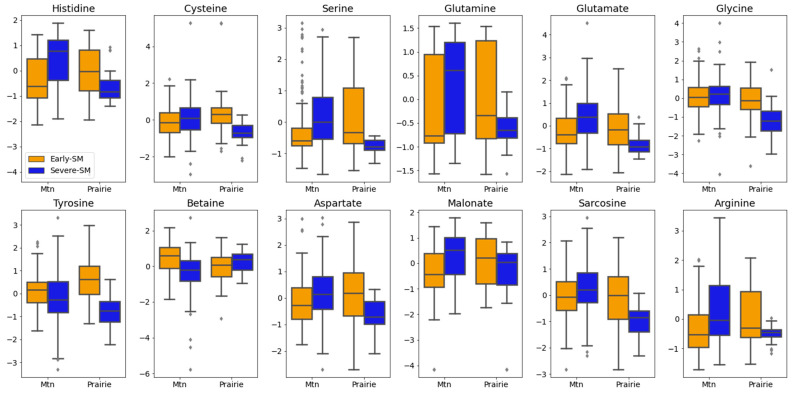
Two-way ANOVA Analysis of Metabolite Patterns in early- versus severe-SM nutritional groups as well as for Wild Bighorn Sheep Residing in Prairie versus Mountain Environments. Box plot representations of metabolite level differences for early-SM (orange/brown) and severe-SM (blue) animal groups captured in mountain (Mtn) versus prairie (Prairie) environments. Normalized metabolite levels for the 12 most significant metabolites (FDR-corrected *p* < 0.0001) display interaction effects between environments and nutritional states. Boxplots depict the interquartile range (IQR) for each metabolite level, with the median value indicated by a horizontal line within each boxplot. Whiskers extend to the furthest point within the 1.5× IQR range, while outliers are marked as individual diamond symbols beyond the whiskers.

In addition to examining the metabolite profiles of the early-SM and severe-SM nutritional groups, we examined the impact of differing habitats, prairie versus mountain environments, on the response of the wild bighorn sheep to nutritional stress. A two-way ANOVA approach was employed to investigate the effects of the environment within the context of early SM and severe SM nutritional stress. This analysis enabled us to establish the statistical significance of metabolite level changes observed within the context of differing habitats, examining adjusted *p*-values to provide a stringent correction for multiple comparisons. This analysis yielded 12 significant metabolites contributing to the separation between early- and severe-SM groups and distinguishing between wild bighorn sheep captured in prairie versus mountain environments (Figure 7). Interestingly, 10 of these metabolites were higher in mean concentration in animals living in prairie versus mountain environments in the early-SM group, except glycine and betaine, whose levels were slightly lower in the prairie early-SM group (Figure 7). The patterns of concentration changes for these same 10 metabolites were reversed (i.e., higher concentration for animals residing in mountain versus prairie habitats) when analyzing the metabolite profiles of the severe-SM animal group. Serum levels of glycine and betaine were higher and lower, respectively, for the mountain severe-SM group compared to the prairie severe-SM animals (Figure 7). The most significant metabolites, as assessed by very small interaction *p*-values, included histidine, cysteine, serine, glutamine, and glutamate. Histidine appeared to be the most significant discriminator between prairie and mountain habitats in early- and severe-SM (*p* = 3.7e-08), underscoring the significant contribution of histidine metabolism to the metabolic adaptation of wild bighorn sheep to different environments and nutritional conditions (Supplementary Table S3). The next most significant metabolites included cysteine (*p* = 1.4e-06) and serine (*p* = 1.8e-06), pointing to significant changes in amino acid and one-carbon metabolism (see discussion below) as part of wild bighorn sheep’s metabolic adaptations to habitat and changes in nutrient availability. Interestingly, several metabolites displayed significant changes associated with environment, i.e., prairie versus mountain, as assessed by environment *p* values (env.p), including sarcosine (env.p = 4.2e-05), glycine (env.p = 1.5e-05) and glutamate (env.p = 0.0012). However, the combined effect of habitat and nutritional status on wild bighorn sheep in prairie versus mountain environments remained the predominant discriminator between early and severe-SM groups, indicating that differing environments and nutritional strains both contribute to the differences in serum metabolite levels quantified here (Supplementary Table S3).

## 4. Discussion

The present study has utilized ^1^H NMR metabolomics to investigate the influence of nutritional status and habitat (i.e., prairie versus mountain) on the serum metabolome of wild bighorn sheep during the transition from winter to spring across different habitats (mountain and prairie) and locations in Wyoming and Montana throughout several winter seasons. This research enabled us to associate different serum metabolite profiles in these animals with distinct nutritional states: early sub-maintenance (early-SM), moderate sub-maintenance (mod-SM), and severe sub-maintenance (severe-SM). These classifications were based on when the animals, captured via helicopter methods, were sampled within the seasonal period and forages were senescent. Examination of 2D PCA (Figure 2A) and PLS-DA (Figure 2B) scores plots resulting from the multivariate statistical analysis of polar serum metabolite levels of the three groups indicated that the serum metabolome of the moderate-SM group cannot be separated from the early-SM and severe-SM groups. This finding suggests that the moderate-SM nutritional state represents a transitional metabolic state between early- and severe-SM. However, due to the small number of serum samples collected from the mod-SM group, data from this group were difficult to reconcile with data from the more numerous serum samples collected from early- and severe-SM animals. Using rigorous statistical metrics, it was decided that removing the moderate-SM group from further comparison between the early- and severe-SM groups was warranted. Analysis of the metabolomics data collected on the early- and severe-SM groups permitted a robust examination of similarities and differences in serum metabolite profiles between the two animal groups. Analysis of the metabolite profiles of early SM versus severe SM revealed significant serum metabolome differences between these two groups of helicopter-captured animals. PLS-DA modeling demonstrated a clear separation of the two groups due to distinct serum polar metabolite profiles (Figure 4A,B), indicating a distinct metabolic response to nutritional stress in these animals. Most significant alterations included differences in serum levels of formate, thymine, glucose, and choline, suggesting that changes in serum concentrations of these metabolites may be valuable indicators of metabolic adaption to nutritional stress in wild bighorn sheep populations (Figure 5). These analyses revealed significant metabolite changes associated with nutritional stress, most notably alterations in metabolites related to metabolic pathways of one-carbon, amino acids, and central carbon metabolism. The significance of these observations is discussed further below.

### 4.1. Wild Bighorn Sheep in Early-SM Versus Severe-SM Nutritional States Can Be Distinguished Based on Alterations in Levels of Metabolites Involved in One-Carbon Metabolism

**Figure 8:**
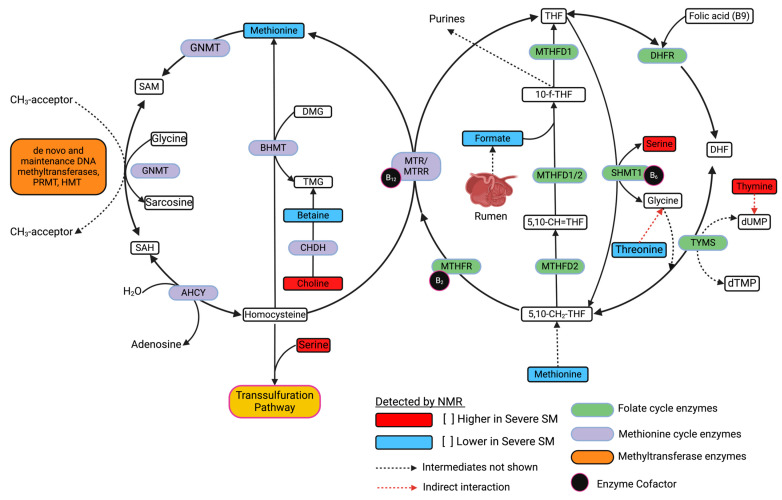
Simplified Overview of One-Carbon Metabolism in Ruminants. This figure highlights pathways involved in one-carbon metabolism in ruminants, emphasizing the interconnection between the folate and methyl cycles and highlighting key enzymes and metabolites detected by NMR in this study. Key enzymes catalyzing essential reactions include Folate Cycle Enzymes (green), Methionine Cycle Enzymes (purple), Transsulfuration Pathway Enzymes (orange), Methyltransferase Enzymes (dark orange), and Propionate Metabolism Enzymes (gray). Metabolites identified and quantified by NMR whose levels are higher (Red) and lower (Blue) in the severe-SM group compared to the early-SM group are also labeled. Intermediate reactions are indicated by dashed lines, and cofactors are shown as black circles. Figure adapted from [38,44]. An expanded version of this figure is presented in Supplementary Figure S4.

Previous research has demonstrated the profound impact of nutritional stress on metabolic pathways utilized by ruminants, including those involved in energy production, amino acid metabolism, and one-carbon metabolism [37,38,39]. One-carbon metabolism, comprising the folate and methionine cycles, plays a pivotal role in cellular function in mammals by providing methyl groups necessary for DNA synthesis, repair, and methylation reactions, as well as for the synthesis of amino acids, polyamines, creatine, and phospholipids [38]. Disruptions of folate and methionine pathways exert significant physiological and developmental consequences for wild ungulates, strengthening the need to understand better the role of these metabolic networks in wild bighorn sheep’s responses to nutritional stress [40]. Findings from this study indicate significant changes in the concentrations of serum metabolites involved in sulfur metabolism and the methionine cycle. These include significant alterations in serum levels of methionine, betaine, choline, and serine (Figure 8). Metabolites involved in the folate cycle, whose levels were significantly altered in early SM versus severe SM animals, included threonine, formate, and thymine (Figure 8). The folate cycle, which is involved in the conversion of dietary folate to 5-methyltetrahydrofolate (5-mTHF), is integral to processes involved in one-carbon metabolism (Figure 8).

Formate was significantly lower in concentration in the severe-SM group. It was one of the strongest discriminators between early-SM and severe-SM animal groups, as revealed by univariate (Figure 6) and multivariate analyses (Figure 5). As illustrated in Figure 8, formate is a key intermediate in one-carbon metabolism, contributing directly to the folate cycle through its conversion to 10-formyl-THF and subsequent regeneration of THF. Additionally, the folate cycle produces 5-methyl-THF, which is essential for the methylation of homocysteine to methionine in the methionine cycle. The significantly lower formate levels observed in severe-SM animals suggest a potential bottleneck in the methionine and folate cycles under severe nutritional stress. This bottleneck could lead to decreased availability of one-carbon units and potentially result in secondary folate deficiency. Moreover, disruption of these cycles may lead to the accumulation of homocysteine, which is toxic to all mammals, including wild ungulates, potentially contributing to increased oxidative stress in severe-SM animals (Figure 8) [41]. Oxidative stress is also a response to chronic nutritional stress, as observed in the metabolic stress experienced by pregnancy and lactation in dairy cows [42]. Response to higher oxidative stress often involves stimulation of the pentose phosphate pathway (PPP) to enhance NADPH production needed to detoxify reactive oxygen species (ROS). Glucose utilization in the PPP, thus, results in diverting glucose from other important metabolic pathways. This scenario would be consistent with the lower serum glucose levels measured in the severe-SM animal group (Supplementary Figure S4) [43].

Methionine metabolism in ruminants is very important, as methionine is widely thought to be a limiting amino acid in ruminant metabolism [45,46]. Methionine supplementation, in the form of rumen-protected methionine (RPM), is currently a topic of great interest in ruminant dairy research, as it has been shown to improve nitrogen utilization, increase rumen microbial crude protein (MCP), and increase volatile fatty acid (VFA) production [47,48].

Nutritional stress in bighorn sheep can also impact one-carbon metabolism as a result of seasonal decreases in the availability of essential vitamins and cofactors, including pyridoxine (vitamin B6), folate (vitamin B9), and/or cobalamin (vitamin B12). These nutrients are specifically required for the proper function of enzymes that catalyze steps of the 1C metabolic cycle (Figure 8). These vitamin deficiencies can also disrupt DNA methylation patterns, altering gene expression and overall health. As methylation reactions are crucial for stress response and DNA repair during nutritional deficits, deficiencies in these vitamins may help explain some of the changes observed in the phenotypes of bighorn sheep throughout the winter season. Our data are consistent with other studies that have demonstrated that maternal nutrition before and during pregnancy can have long-term effects on offspring’s health through epigenetic modifications [38]. In domestic sheep, restricted dietary intake of methionine and vitamin B12 has been associated with obesity, insulin resistance, and hypertension in adult offspring, highlighting the importance of these nutrients in early development and their potential to program metabolic outcomes [49].

### 4.2. Alterations in Amino Acid Metabolism Reflect Nutritional Stress and Possible Energy Deficits in Wild Bighorn Sheep

In addition to alterations in the levels of metabolites involved in one-carbon metabolism, our analysis identified significant changes in the serum concentrations of several amino acids when comparing early- versus severe-SM nutritional states of wild bighorn sheep. Significant differences in serum concentrations were measured for glutamine, threonine, tyrosine, methionine, valine, serine, and arginine (Figure 8 and Figure S4). These amino acids are crucial in various metabolic processes, including protein synthesis, nitrogen cycling, immune function, and energy production [50]. Glutamine is pivotal in nitrogen transport and immune function. Increased levels in the severe-SM group may indicate a compromised ability to maintain nitrogen balance and/or altered immune responses following nutritional stress. Similar findings have been observed in other studies, where nutritional deprivation leads to altered plasma glutamine levels, which are correlated with impaired immune function and increased susceptibility to infections [51,52]. As wild bighorn sheep are highly susceptible to epizootic pneumonia infections, the effects of nutritional stress on their immune system are not to be discounted. Indeed, recent work with the same wild bighorn sheep herds that have been the focus of this study supports the idea that the underlying nutritional state of the animals is a major factor when considering susceptibility to pneumonia infections [53,54]. These recent studies have also demonstrated a direct link between limiting nutritional status and energetic costs associated with pathogen infection [53,54].

Threonine, serine, histidine, and methionine are essential for protein synthesis and methyl group transfer reactions and are rarely used for gluconeogenesis in ruminants [55]. Higher serum levels of serine and histidine in the severe-SM group might reflect increased muscle protein catabolism in these animals. Serum levels of these two amino acids could, thus, be indicators of muscle atrophy and weakened physical condition of the severe-SM wild bighorn sheep. Lower serum threonine levels observed in the severe-SM groups are consistent with research findings on domestic sheep, where threonine deficiency has been linked to reduced growth rates and protein catabolism [56]. Histidine, which is higher in concentration in the severe-SM group, has also been shown to suppress food intake in non-ruminant species through its conversion to histamine [57]. Our observed elevated serum levels could, thus, reflect the role of histidine in mediating food intake and the nutritional adaption of the severe-SM group to lower forage availability. Serine, which is slightly higher in concentration in the severe-SM group relative to the early-SM group, is utilized in methyl group transfer reactions, further providing evidence of metabolic perturbations associated with amino acid (AA) metabolism and the one-carbon cycle [55].

The branched-chain amino acids (BCAAs), valine and isoleucine, are essential components of all mammalian diets, including ruminants, as these cannot be synthesized de novo. BCAAs are involved in many cellular processes in ruminants, including regulating rumen fermentation, intestinal development and function, mammary development, skeletal muscle growth, and adipose tissue homeostasis [58]. BCAAs also provide carbon and nitrogen for the synthesis of non-essential amino acids (NEAAs), contributing directly to energy homeostasis by providing precursors for the synthesis of TCA cycle intermediates [59]. Lower serum levels of valine (Figure 5B and Figure 6) and isoleucine (Figure 6) in severe-SM wild bighorn sheep are consistent with BCAAs being used for energetics (ATP) homeostasis during periods of reduced nutrient intake, as has been demonstrated in other ruminants [60,61]. Further exploring the impact of AA metabolism alterations would be a valuable future endeavor, as it would strengthen our understanding of how seasonal changes affect the diet and nutritional state of wild ruminants.

### 4.3. Severe Nutritional Stress Possibly Alters Choline Metabolism, TCA Cycle Activity, Nucleotide Metabolism, and Nitrogen Detoxification in Wild Bighorn Sheep

Choline is an essential dietary nutrient in ruminants and plays a significant role in lipid metabolism. It is a necessary constituent of phospholipids, including phosphatidylcholine (PtCho), an essential element of the neurotransmitter acetylcholine (Acho) [62]. Choline is vital for lipid transport, as PtCho is necessary for synthesizing very low-density lipoproteins (VLDL), which are the primary source of lipids for extrahepatic tissues in ruminants [63]. Additionally, choline is an essential source of transferrable methyl groups in several anabolic processes as it serves as a precursor for the synthesis of betaine [62]. Elevated serum levels of choline were measured in contrast to the decreased levels of both betaine and methionine in the severe-SM group (Figure 8). This suggests several possibilities, including impairment of methyl transfer pathways as a result of inefficient conversion of choline to betaine or an excess demand for methyl donors, reduced liver function affecting methionine and betaine synthesis in nutritionally stressed animals, dietary imbalances due to seasonal changes, or elevated homocysteine levels resulting from increased serum choline levels (Supplementary Figure S4).

Significant differences in the serum concentration of thymine, a pyrimidine base, were observed in early-SM (lower) versus severe-SM (higher). In ruminants, as with most animals, nucleotide metabolism is closely connected to overall health as rapidly dividing cells, such as those in the gut lining and immune system, require a constant supply of nucleotides [64]. In addition, malonate was found to be higher in concentration in the severe SM group (Figure 6), which suggests that as a competitive inhibitor of succinate dehydrogenase (SDH), serum levels of malonate may reflect alterations in TCA cycle function in the severe-SM group. Alterations in TCA cycle activity are also consistent with our observation of elevated serum succinate levels in the severe-SM group (Figure 8). This could be due to increased reactive oxygen species (ROS) production and overall metabolic stress experienced by the severe-SM animals [65]. Arginine, found to be elevated in the serum of the severe-SM group (Figure 4 and Figure 5), is a key component of nitric oxide production, polyamine synthesis, and the urea cycle in ruminants. Arginine plays a crucial role in ammonia detoxification via the urea cycle and arginase-catalyzed hydrolysis of arginine to urea and ornithine [41]. We postulate that elevated serum levels of arginine may reflect higher needs for increased ammonia detoxification resulting from increased excretion of excess nitrogen associated with increased muscle catabolism in severe-SM wild bighorn sheep compared to their early-SM counterparts.

### 4.4. Differences in the Metabolic Response of Early-SM and Severe-SM Wild Bighorn Sheep Are Distinguished by the Capture Environment

Two-way ANOVA analysis was employed to assess whether metabolite differences observed between early SM and severe SM were also impacted by the environment, i.e., prairie versus mountain habitats where the wild bighorn sheep were captured for this study. This analysis revealed that 10 out of 12 most significant metabolites were higher in concentrations in prairie-captured wild bighorn sheep during early SM (excluding betaine and glycine). This pattern was reversed in the severe-SM group, with higher concentrations found in mountain-captured wild bighorn sheep (excluding betaine) (Figure 8). This shift in serum metabolite level patterns indicates a dynamic metabolic response to nutritional stress and environmental conditions. For instance, the metabolite patterns observed for histidine, cysteine, and serine indicated significant environmental and nutritional interaction effects, underscoring the importance of the environment in metabolic adaptation processes. Histidine was particularly noteworthy, with the most significant interaction effect (*p* = 3.7e-08), followed by cysteine (*p* = 1.4e-06) and serine (*p* = 1.8e-06) (Figure 8). The contrasting metabolic patterns suggest that prairie environments may offer particular advantages, imparting less severe nutritional stress during early winter months, possibly due to the availability of more abundant or higher quality nutrients in a prairie environment [6] and greater and longer access to forage due to generally lower snowpack in prairie habitats compared to snowpack at higher elevation mountain environments. Conversely, mountain environments might provide a degree of metabolic resilience when animals experience more severe nutritional stress, potentially reflecting the physiological adaptations of wild bighorn sheep to mountainous environments where they are frequently found [3].

This metabolic flexibility is crucial for survival, allowing wild bighorn sheep to adapt to varying conditions by modulating critical metabolic pathways. Identification of metabolites such as histidine, cysteine, serine, glutamine, and glutamate as significant based on their interaction *p*-values further supports the premise that environmental and nutritional factors jointly influence the nutritional status (early-SM and severe-SM) of wild bighorn sheep. This analysis underscores the interplay between environmental conditions and nutritional stress in modulating wild bighorn sheep’s metabolic responses and adaptation. A deeper understanding of the crosstalk between environment and metabolic responses is vital to inform conservation efforts and to help guide strategies to enhance the resilience and sustainability of bighorn sheep populations in diverse habitats.

Understanding the intricacies of one-carbon, amino acid, and central metabolism in wild bighorn sheep has significant implications for the conservation and management of these animals moving forward. A summary of the metabolite changes observed as they relate to biochemical pathways and physiological processes is shown in Supplementary Table S4. Ensuring these animals have adequate access to quality nutrition, particularly during critical periods such as gestation and early development, is essential for maintaining health and population stability. Conservation strategies may include monitoring dietary intake to better identify possible deficiencies that could lead to adverse health outcomes. Additionally, the role of 1C metabolism in epigenetic regulation underscores the potential for transgenerational effects of nutritional stress [66]. Effective management practices may consider the immediate dietary needs of bighorn sheep and the long-term impacts of nutritional stress on their offspring’s health and survival.

## 5. Conclusions

This analysis underscores the value of quantifying serum metabolome changes in wild bighorn sheep to enhance our understanding of critical metabolic shifts accompanying varying degrees of nutritional stress. Overall, the metabolite alterations characterized in this study have provided new knowledge about the complex metabolic adaptations of wild bighorn sheep when experiencing different degrees of sub-maintenance nutritional stress. Marked differences in concentrations of key metabolites between early sub-maintenance and severe sub-maintenance animal groups have provided valuable insights into biochemical pathways and cellular mechanisms underpinning the physiological responses of these animals to nutritional challenges. Our findings emphasize the importance of ensuring adequate dietary intake for bighorn sheep, particularly during critical seasonal periods.

In conclusion, this study has identified and quantified 49 water-soluble metabolites in the serum samples of large numbers of wild bighorn sheep living in different environments. A series of thorough and rigorous univariate and multivariate analyses of serum metabolite profiles identified 15 metabolites that were strong discriminators of severe versus early sub-maintenance nutrition. Herein, we demonstrate that quantifying the levels of metabolites in the sera of wild bighorn are robust indicators of the nutritional stress experienced by wild bighorn sheep in different environments. The work presented here highlights the complex metabolic adaptations of wild bighorn sheep to nutritional stress, with significant implications for their conservation and management. Future research is needed to explore further the biological information contained in quantifying alterations in metabolite levels in wild bighorn sheep. The current work has demonstrated the potential of metabolomics as a powerful analytical approach to define reliable biological indicators of nutritional status, which could be leveraged to guide more effective conservation strategies to ensure the robust, long-term survival of these iconic animals.

## Figures and Tables

**Table 1:** List of Metabolites whose Levels Differ Significantly between Early- and Severe-SM groups, as Identified by Volcano Plot Analysis. This table highlights significant metabolites based on the following threshold parameters: |log_2_ fold change| > 1 and -log_10_ *p*-value comparing the ratio of metabolite levels in early- versus severe-SM nutritional groups. Columns indicate FC: Fold change, i.e., ratios of metabolite levels, indicating increases (>1) or decreases (<1) in early-SM versus severe-SM groups; log_2_ (FC): log_2_ transformed fold change; positive values indicate increased concentrations, negative values decreased concentrations in early- versus severe-SM groups., *p*-adjusted: adjusted *p*-value for multiple comparisons, obtained from Wilcoxon rank tests and reporting on metabolites with *p*-values < 0.05; and −log_10_ (p): negative log_10_ of the adjusted *p*-value, highlighting significant metabolite level differences between the two animal groups.

Metabolite	FC	log_2_(FC)	p.ajusted	−log_10_(p)
Formate	5.1	2.3	1.33e-17	16.9
Glucose	2.8	1.5	2.34e-17	16.6
Thymine	0.3	−1.9	1.25e-15	14.9
Valine	3.2	1.7	2.40e-11	10.6
Creatinine	0.3	−1.6	4.65e-11	10.3
Choline	0.4	−1.4	1.05e-10	10
Threonine	2.5	1.3	2.61e-10	9.6
Betaine	2	1	2.86e-06	5.5
Dimethyl sulfone	2	1	3.26e-06	5.5
2-Oxoisocaproate	2.2	1.2	2.04e-04	3.7

## Data Availability

Data will be deposited in the Metabolomics Workbench data repository as study ST003675 for public access following acceptance of the manuscript for publication. The data will also be accessible to scientists and investigators via direct request to the authors. All code used for running experiments, model fitting, and plotting are available on a GitHub repository at https://github.com/Galenosheastone/Copie_Group_Metabolomics_Stats_Tools_2025 (accessed on 20 February 2025). All code is also accessible to scientists and investigators via direct request to the authors.

## Supplementary Materials

The following supporting information can be downloaded at https://www.mdpi.com/article/10.3390/metabo15030154/s1, Figure S1: Impact of Excluding Moderate (“Mod”) Submaintenance Group on PCA, Kolmogorov-Smirnov (KS) Metabolite Test Results, and Overall Dataset Distributions; Table S1: Summary of Animal Measurements by Herd and Nutritional State; Figure S2: Comparison of PCA and Density Plots for Original and Modified Dataset; Figure S3: Summary of PLS-DA metrics; Table S2: Differential Metabolite Levels Between Early sub-maintenance (Early-submaint) and Severe sub-maintenance (Severe-submaint) Groups; Table S3: Two-way ANOVA results: environmental effect *p*-value, nutritional effect *p*-value and interaction *p*-value; Figure S4: Simplified schematic of One-Carbon Metabolism and Simplified Energy Metabolism in Ruminants; Table S4: Summary of the 15 significant metabolites considered to be strong discriminators of distinct nutritional states in wild bighorn sheep.
