# Supplementary material for: Polar Metabolite Profiles Distinguish Between Early and Severe Sub-Maintenance Nutritional States of Wild Bighorn Sheep
Source: Metabolites. 2025 Feb 24;15(3):154. doi: 10.3390/metabo15030154 (PMC11943576; doi:10.3390/metabo15030154)
Supplement: Supplementary file 1 [file metabolites-15-00154-s001.zip › metabolites-3467473-supplementary.pdf]

Supplementary Information

Title: Polar Metabolite Profiles Distinguish Between Early and Severe Sub-maintenance Nutritional States of Wild Bighorn Sheep.

Authors: O'Shea-Stone et al. (2025)

A.

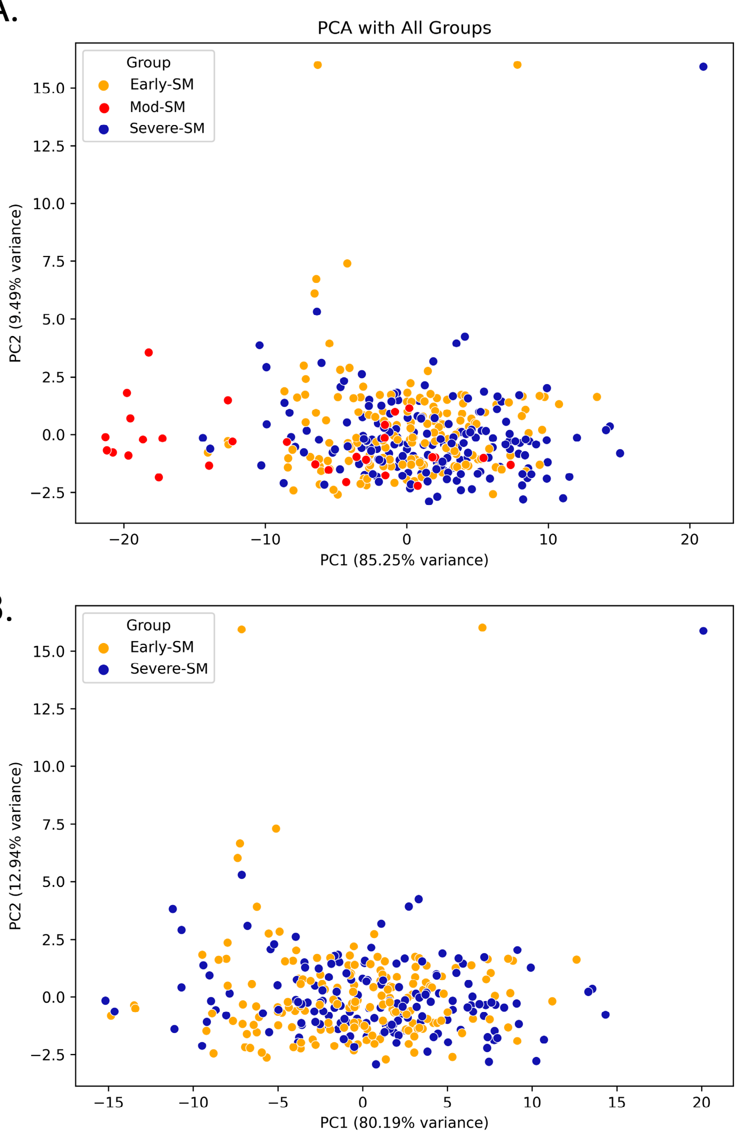

C. Kolmogorov-Smirnov (KS) Test Results

Metabolite	KS Statistic	p-value
**OVERALL**	**0.0042**	**0.99**
2-Oxoisocaproate	1.09E-02	1.00
3-Hydroxybutyrate	1.48E-02	1.00
3-Methyl-2-oxovalerate	1.28E-02	1.00
Acetate	2.68E-02	1.00
Alanine	1.69E-02	1.00
Allantoin	2.33E-02	1.00
Arginine	2.05E-02	1.00
Asparagine	3.35E-02	0.98
Aspartate	1.14E-02	1.00
Betaine	5.88E-02	0.53
Choline	2.44E-02	1.00
Creatine	2.12E-02	1.00
Creatine phosphate	2.13E-02	1.00
Creatinine	1.99E-02	1.00
Cysteine	2.90E-02	1.00
Dimethyl sulfone	5.91E-02	0.52
Dimethylamine	3.61E-02	0.96
Formate	1.79E-02	1.00
Fructose	4.38E-02	0.86
Glucose	4.58E-02	0.82
Glutamate	1.17E-02	1.00
Glutamine	3.17E-02	0.99
Glycerol	3.04E-02	0.99
Glycine	4.38E-02	0.85
Hippurate	1.24E-02	1.00
Histidine	3.51E-02	0.97
Isoleucine	1.42E-02	1.00
Lactate	4.34E-02	0.86
Leucine	2.48E-02	1.00
Lysine	2.36E-02	1.00
Malonate	3.34E-02	0.98
Methionine	1.08E-02	1.00
O-Acetylcarnitine	2.51E-02	1.00
Phenylalanine	3.77E-02	0.95
Proline	1.92E-02	1.00
Propionate	8.98E-03	1.00
Pyruvate	3.12E-02	0.99
Sarcosine	1.71E-02	1.00
Serine	1.12E-02	1.00
Succinate	3.92E-02	0.93
Threonine	4.55E-02	0.82
Thymine	2.50E-02	1.00
Trimethylamine N-oxide	1.11E-02	1.00
Tryptophan	3.83E-02	0.94
Tyrosine	2.61E-02	1.00
Urea	2.04E-02	1.00
Valine	2.56E-02	1.00

Figure S1: Impact of Excluding Moderate ("Mod") Submaintenance Group on PCA, Kolmogorov-Smirnov (KS) Metabolite Test Results, and Overall Dataset Distributions. Results illustrate the results of Principal Component Analysis (PCA) and Kolmogorov-Smirnov (KS) tests for the metabolomics data comparing nutritional stress conditions upon inclusion or exclusion of the "Mod-SM" group. **S1.A.** PCA plot with all groups (Early-SM, orange; Mod-SM, red; and Severe-SM, blue) shows the clustering of metabolite profiles along the first two principal components (PC1 and PC2). **S1.B.** PCA plot excluding the "Mod-SM" group, displaying only Early-SM and Severe-SM groups. **S1.C.** A table summarizing the KS test results for both datasets (OVERALL), and individual metabolites, showing the KS statistic and p-

value for each when comparing the full dataset to the dataset without "Mod-SM." The KS test evaluates the distributional differences between metabolite concentrations in the original dataset (including Mod-SM) and the modified dataset (excluding Mod-SM). The p-values indicate that, overall, there is no significant distributional differences between the two datasets in the overall comparison, or in any single metabolite (significant difference p-values < 0.05).

Table S1. Summary of Animal Measurements by Herd and Nutritional State. Provides a summary of the animals included in the study, listing their respective herds, the number of measurements taken, and their classification into different nutritional states, “Early-submaint” (early-SM) and “Severe-submaint" (severe-SM).

Animal Id	Measure Count	Herd	Nutritional State
1	4	Dubois	['Early-submaint' 'Severe-submaint']
20	4	Dubois	['Early-submaint' 'Severe-submaint']
18	4	Dubois	['Early-submaint' 'Severe-submaint']
16	4	Dubois	['Early-submaint' 'Severe-submaint']
15	4	Dubois	['Early-submaint' 'Severe-submaint']
13	4	Dubois	['Early-submaint' 'Severe-submaint']
25	4	Jackson	['Early-submaint' 'Severe-submaint']
11	4	Dubois	['Early-submaint' 'Severe-submaint']
26	4	Jackson	['Early-submaint' 'Severe-submaint']
27	4	Jackson	['Early-submaint' 'Severe-submaint']
8	4	Dubois	['Early-submaint' 'Severe-submaint']
6	4	Dubois	['Early-submaint' 'Severe-submaint']
29	4	Jackson	['Early-submaint' 'Severe-submaint']
31	4	Jackson	['Early-submaint' 'Severe-submaint']
21	4	Jackson	['Early-submaint' 'Severe-submaint']
28	3	Jackson	Severe-submaint
23	3	Jackson	Severe-submaint
30	3	Jackson	Severe-submaint
22	3	Jackson	Severe-submaint
52	3	Jackson	['Early-submaint' 'Severe-submaint']
14	3	Dubois	['Early-submaint' 'Severe-submaint']
12	3	Dubois	['Early-submaint' 'Severe-submaint']
10	3	Dubois	Severe-submaint
5	3	Dubois	['Early-submaint' 'Severe-submaint']
3	3	Dubois	['Early-submaint' 'Severe-submaint']
49	3	Jackson	['Early-submaint' 'Severe-submaint']
51	2	Jackson	Severe-submaint
47	2	Jackson	['Early-submaint' 'Severe-submaint']
50	2	Dubois	Severe-submaint
60	2	Jackson	['Early-submaint' 'Severe-submaint']
61	2	Dubois	['Early-submaint' 'Severe-submaint']
62	2	Dubois	['Early-submaint' 'Severe-submaint']
2	2	Dubois	Severe-submaint
4	2	Dubois	['Early-submaint' 'Severe-submaint']
24	2	Jackson	Severe-submaint
17	2	Dubois	['Early-submaint' 'Severe-submaint']
9	2	Dubois	Severe-submaint

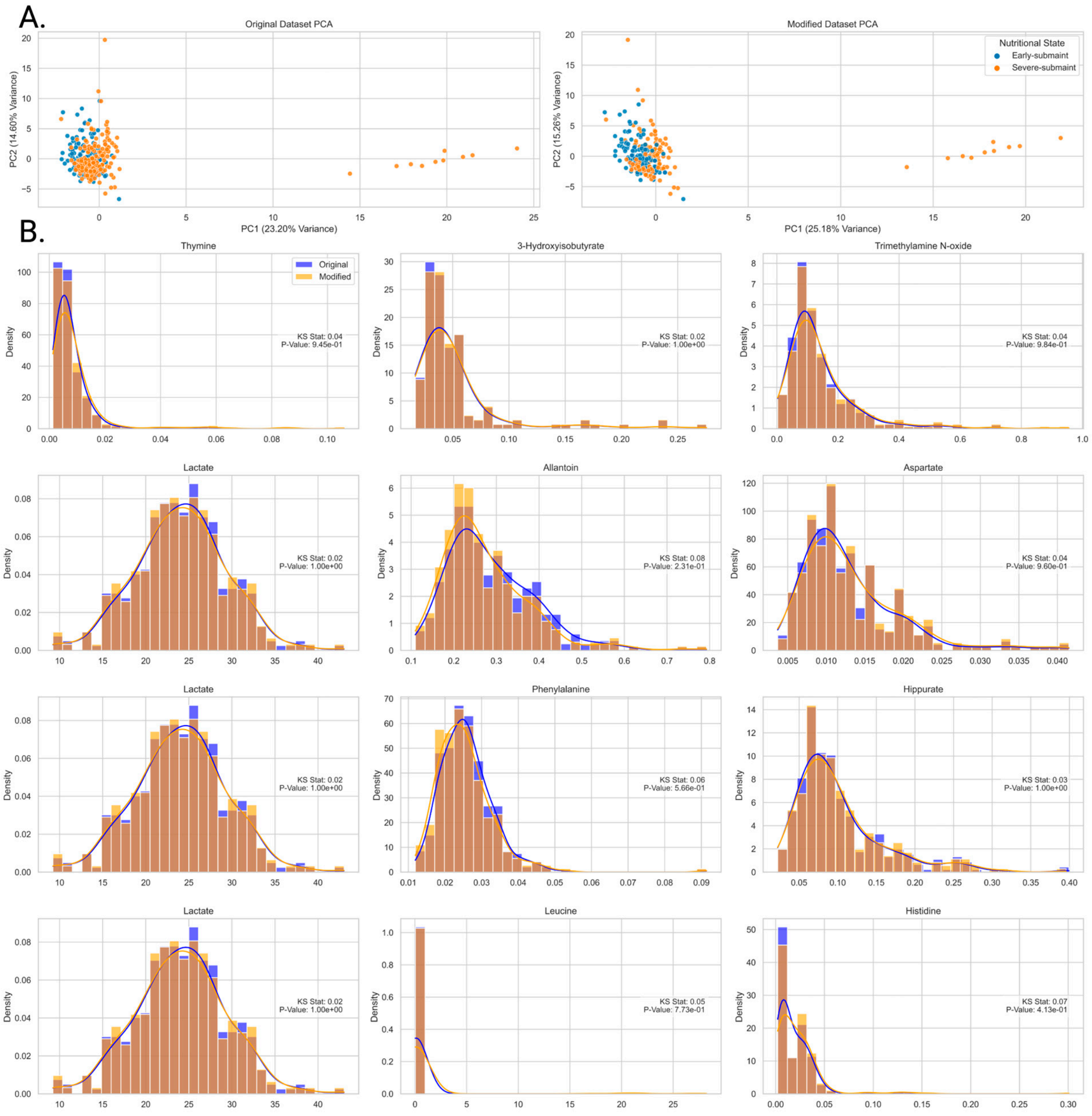

Figure S2. Comparison of PCA and Density Plots for Original and Modified Dataset. Principal Component Analysis (PCA) and density plots of a subset of 10 metabolites from the original dataset (including repeated measurements) and the modified dataset (excluding repeated measurements). The PCA plots (**A**) show the variance explained by each principal component, and the density plots (**B**) depict the distribution of metabolite concentrations. The x-axis in the histograms is the relative concentration. Analysis confirms that removing repeated measurements has minimal impact on the overall distribution and pattern of the metabolite profiles.

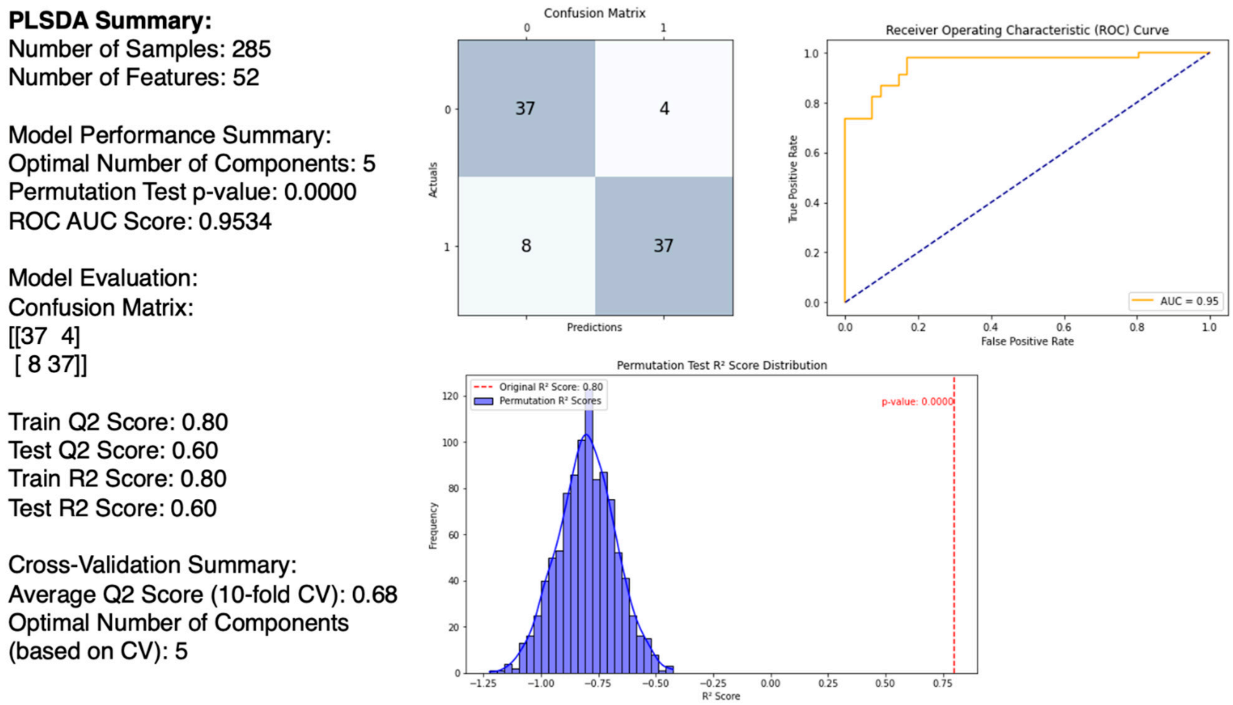

Figure S3: Summary of PLS-DA metrics. Metrics associated with the PLS-DA model used to distinguish between the early-submaintenance (early-SM) and severe-submaintenance (severe-SM) nutritional states. The PLS-DA model was cross-validated, and the metrics displayed include training and test R^2 and Q^2 values, confusion matrix results, ROC value, cross-validated Q2 average and permutation test results.

Table S2: Differential Metabolite Levels Between Early-submaint and Severe-submaint Groups. Table comparing metabolite levels between the Early-submaint (early-SM) and Severe-submaint (severe-SM) groups, highlighting significant differences from Mann-Whitney U test, expressed as standardized z-scores derived from log-transformation and autoscaling. Columns include each metabolite's name, average levels in both groups, the level difference, and the adjusted p-value for statistical significance. Positive level differences indicate higher metabolite concentrations in the early-SM group, while negative values indicate higher levels in the severe-SM group. Adjusted p-values were calculated using the Benjamini-Hochberg false discovery rate method, with values below 0.05 indicating statistical significance.

Metabolite	Level_Early-submaint	Level_Severe-submaint	Level_Difference	Adjusted P-value
Formate	0.51	-0.55	1.06	1.53E-18
Glucose	0.42	-0.47	0.89	5.20E-14
Thymine	-0.39	0.43	-0.82	3.10E-12
Choline	-0.36	0.40	-0.76	2.36E-09
Valine	0.35	-0.38	0.72	1.85E-09
Threonine	0.31	-0.34	0.65	4.51E-08
Tyrosine	0.29	-0.32	0.60	4.14E-07
Pyruvate	0.28	-0.31	0.59	1.34E-06
Betaine	0.28	-0.31	0.59	2.78E-06
Creatinine	-0.27	0.29	-0.56	1.21E-08
Histidine	-0.23	0.26	-0.49	3.49E-05
Dimethylamine	0.23	-0.26	0.49	2.50E-04
Dimethyl sulfone	0.23	-0.25	0.48	1.32E-05
Asparagine	-0.20	0.22	-0.43	8.76E-03
2-Oxoisocap.	0.19	-0.21	0.41	3.66E-03
Malonate	-0.19	0.21	-0.40	7.54E-04
Fructose	-0.19	0.21	-0.40	2.55E-03
3-M-2-Ovalerate	0.19	-0.21	0.40	3.66E-03
Methionine	0.18	-0.19	0.37	1.76E-03
Arginine	-0.15	0.16	-0.31	2.55E-03
Isoleucine	0.14	-0.15	0.30	2.17E-02
Alanine	0.14	-0.15	0.30	2.17E-02
3-Hybutyrate	-0.14	0.15	-0.29	3.08E-02
Glutamine	-0.11	0.13	-0.24	2.50E-02

Table S3: Two-way ANOVA results: environmental effect p-value, nutritional effect p-value and interaction p-value. This table presents the results of a two-way ANOVA analysis assessing the effects of nutritional state and capture environment on metabolite concentrations. The table includes p-values for the environmental effect, nutritional effect, and their interaction, identifying metabolites significantly impacted by these factors.

	Environment_p	NutritionalState_p	Interaction_p
**Histidine**	ns	2.39E-05	3.71E-08
**Cysteine**	ns	ns	1.37E-06
**Serine**	ns	ns	1.84E-06
**Glutamine**	ns	1.80E-02	2.05E-06
**Glutamate**	1.21E-03	ns	5.33E-06
**Glycine**	1.51E-05	ns	9.48E-06
**Tyrosine**	ns	2.05E-06	1.15E-05
**Betaine**	ns	1.37E-06	3.12E-05
**Aspartate**	ns	ns	3.42E-05
**Malonate**	ns	3.64E-04	4.23E-05
**Sarcosine**	4.23E-05	ns	5.59E-05
**Arginine**	ns	9.61E-03	8.58E-05

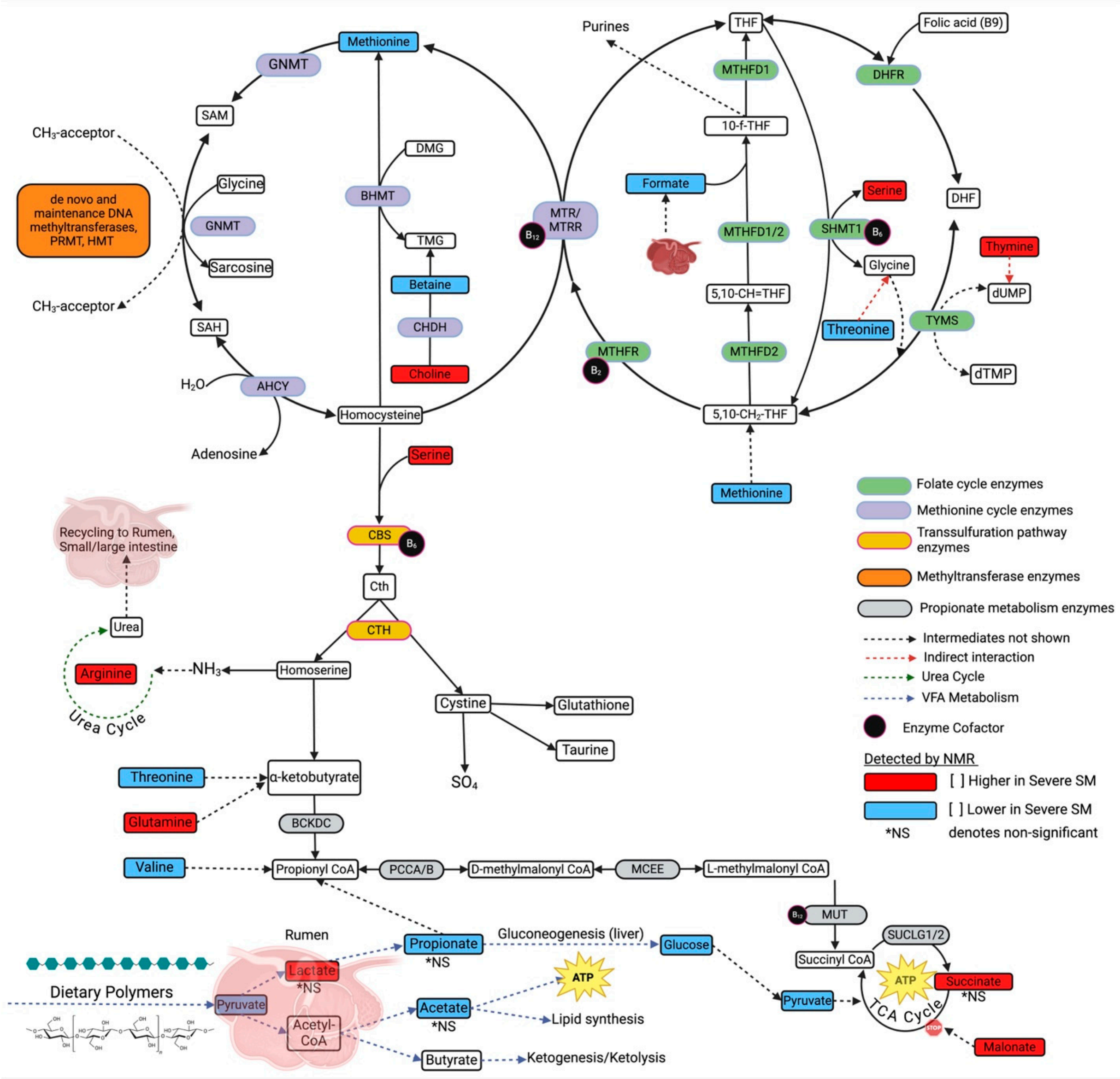

Figure S4: Simplified schematic of One-Carbon Metabolism and Simplified Energy Metabolism in Ruminants. This figure highlights pathways involved in one-carbon metabolism in ruminants, emphasizing the interconnection between the folate and methyl cycles, and highlighting key enzymes and metabolites detected by NMR in the study. Key enzymes catalyzing essential reactions include: Folate Cycle Enzymes (green); Methionine Cycle Enzymes (purple); Transsulfuration Pathway Enzymes (orange); Methyltransferase Enzymes (dark orange); and Propionate Metabolism Enzymes (gray). Metabolites identified and quantified by NMR whose levels are higher (Red) and lower (Blue) in the severe-SM group compared to the early-SM group are also labeled. Intermediate reactions are indicated by dashed lines and cofactors are shown as black circles. Additional pathways highlighting the fate of metabolites involved in cellular energetics include: the urea cycle (green dashed line); the TCA Cycle; and lipid metabolism including volatile fatty acids (VFA) (light blue dashed line). Figure adapted from [38,44].

Table S4: Summary of the 15 metabolites identified with VIP scores >1.0, and considered to be strong discriminators of distinct nutritional states in wild bighorn sheep. The metabolites are listed with their respective concentration trends (positive +, or negative -) in Early-SM and Severe-SM groups, as well as their associated generic biochemical pathways and basic associated physiological effects.

Metabolite	Early-SM	Severe-SM	Biochemical Pathway(s)	Physiological Effect/Process
Formate	+	-	One-carbon metabolism	Immune function, methylation
Thymine	-	+	Nucleotide metabolism	DNA repair, immune modulation
Glucose	+	-	Central carbon metabolism	Energy metabolism
Choline	-	+	Lipid metabolism, methylation	Fat transport, methyl group donor
Glutamine	-	+	Amino acid metabolism	Immune response, nitrogen transport
Threonine	+	-	One-carbon metabolism	Protein synthesis
Histidine	-	+	Amino acid metabolism	Protein degradation, immune function
Tyrosine	+	-	Amino acid metabolism	Neurotransmitter precursor
Methionine	+	-	One-carbon metabolism	Methylation, protein synthesis
Valine	+	-	Amino acid metabolism (BCAA)	Muscle growth, energy source
Betaine	+	-	One-carbon metabolism	Methyl group donor, osmoregulation
Serine	-	+	Amino acid metabolism	Protein synthesis, methylation
Pyruvate	+	-	Central carbon metabolism	Energy production
Malonate	-	+	TCA cycle inhibition	Energy production inhibition
Arginine	-	+	Amino acid metabolism	Nitric oxide production, detoxification
